# Cardiac autonomic control in Rett syndrome: Insights from heart rate variability analysis

**DOI:** 10.3389/fnins.2023.1048278

**Published:** 2023-03-20

**Authors:** Ramona Cordani, Eleonora Tobaldini, Gabriel Dias Rodrigues, Donatella Giambersio, Marco Veneruso, Lorenzo Chiarella, Nicola Disma, Elisa De Grandis, Edgar Toschi-Dias, Ludovico Furlan, Angelica Carandina, Giulia Prato, Lino Nobili, Nicola Montano

**Affiliations:** ^1^Department of Neuroscience, Rehabilitation, Ophthalmology, Genetics, Maternal and Child Health, University of Genoa, Genoa, Italy; ^2^Unit for Research & Innovation in Anesthesia, IRCCS Istituto Giannina Gaslini, Genova, Italy; ^3^Department of Clinical Sciences and Community Health, University of Milan, Milan, Italy; ^4^Department of Internal Medicine, Fondazione IRCCS Ca’ Granda, Ospedale Maggiore Policlinico, Milan, Italy; ^5^Child Neuropsychiatry Unit, IRCCS Istituto Giannina Gaslini, Genova, Italy; ^6^Department of Basic Medical Sciences, Neuroscience and Sense Organs, University of Bari “Aldo Moro”, Bari, Italy; ^7^Health Psychology Program, Methodist University of São Paulo, São Paulo, Brazil; ^8^Psychology, Development and Public Policy Program, Catholic University of Santos, São Paulo, Brazil

**Keywords:** Rett syndrome (RTT), dysautonomia, methyl-CpG-binding protein 2 (MECP2), cardiac autonomic control, autonomic nervous system, heart rate variability (HRV)

## Abstract

Rett syndrome (RTT) is a rare and severe neurological disorder mainly affecting females, usually linked to methyl-CpG-binding protein 2 (MECP2) gene mutations. Manifestations of RTT typically include loss of purposeful hand skills, gait and motor abnormalities, loss of spoken language, stereotypic hand movements, epilepsy, and autonomic dysfunction. Patients with RTT have a higher incidence of sudden death than the general population. Literature data indicate an uncoupling between measures of breathing and heart rate control that could offer insight into the mechanisms that lead to greater vulnerability to sudden death. Understanding the neural mechanisms of autonomic dysfunction and its correlation with sudden death is essential for patient care. Experimental evidence for increased sympathetic or reduced vagal modulation to the heart has spurred efforts to develop quantitative markers of cardiac autonomic profile. Heart rate variability (HRV) has emerged as a valuable non-invasive test to estimate the modulation of sympathetic and parasympathetic branches of the autonomic nervous system (ANS) to the heart. This review aims to provide an overview of the current knowledge on autonomic dysfunction and, in particular, to assess whether HRV parameters can help unravel patterns of cardiac autonomic dysregulation in patients with RTT. Literature data show reduced global HRV (total spectral power and R-R mean) and a shifted sympatho-vagal balance toward sympathetic predominance and vagal withdrawal in patients with RTT compared to controls. In addition, correlations between HRV and genotype and phenotype features or neurochemical changes were investigated. The data reported in this review suggest an important impairment in sympatho-vagal balance, supporting possible future research scenarios, targeting ANS.

## Introduction

Rett syndrome (RTT), OMIM #312750, is a rare neurological disorder characterized by severe cognitive, social, and physical impairments, mainly affecting females (Jeffrey et al., 2010; Tascini et al., 2022), being the second most prevalent genetic cause of intellectual disability in girls (incidence of 1/10,000) (Borloz et al., 2021). First described in the 1960s by Andreas Rett (Rett, 1966; Jeffrey et al., 2010), the syndrome was subsequently clinically characterized by Hagberg in classical and atypical RTT forms (Hagberg et al., 1983; Hagberg, 2002; Tascini et al., 2022). Amir et al. (1999) identified the genetic cause of RTT as a loss of function mutation in the gene Methyl-CpG-binding protein 2 (MECP2). More than 800 *de novo* mutations of MECP2 gene are responsible for 95% cases of classical RTT (Tascini et al., 2022) and 50–70% of AR in which other genes, such as cyclin-dependent kinase-like 5 (CDKL5) and forkhead box G1 (FOXG1) gene, are also involved (Guerrini and Parrini, 2012; Tascini et al., 2022). Nevertheless, RTT continues to be primarily a clinical diagnosis, as MECP2 mutations are neither necessary nor sufficient for the diagnosis (Ramirez et al., 2020). Patients with RTT present a distinctive clinical course and key clinical features, including autonomic dysfunction. This literature review aims to overview the current knowledge on autonomic dysfunction in patients with RTT and its relationship with the increased incidence of sudden death in these patients compared to the general population. In particular, we intend to assess whether heart rate variability parameters can help unravel patterns of autonomic dysregulation in patients with RTT.

## Rett syndrome: Clinical features

The classical RTT onset is between the 6th and 18th months in subjects with an apparently normal development during the pre/perinatal period as well as the first months of life (Hagberg, 2002). According to the 2010 revised criteria, regression is a key element for diagnosis along with four main criteria (Hagberg, 2002): (1) partial or complete loss of acquired purposeful hand skills, (2) partial or complete loss of acquired spoken language, (3) gait abnormalities (impaired -dyspraxic- or absence of ability), (4) stereotypic hand movements (such as hand wringing/squeezing, clapping/tapping, mouthing, and washing/rubbing automatism) (Jeffrey et al., 2010). Four stages compose the typical clinical course of Rett syndrome (Hagberg, 2002; Cosentino et al., 2019; Tascini et al., 2022). The first phase (6–18 months) is characterized by a slowdown in the development and is followed by a second stage (1–4 years) distinguished by skill regression, profound loss of acquired communication and hand use skills, and autistic-like behavior. Ataxia and apraxia could occur at this stage, and in many cases, patients develop microcephalia. Irritability is extremely common. A pseudo-stationary stage denotes the third phase (2–10 years) with recovery or motor stabilization, improved interaction, and reduction in irritability. However, during this period, epileptic seizures may occur. The fourth phase (>10 years) is characterized by a progressive decrease in motility, parkinsonism, joint contractures, scoliosis, and other orthopedic issues. However, epilepsy may improve.

During the last part of their life, patients undergo a worsening motor performance (Hagberg, 2002; Cosentino et al., 2019; Tascini et al., 2022). “Atypical or variant” Rett is defined by the presence of many of the clinical characteristics of RTT but not completely satisfied. These variants range from milder conditions with a later age of onset to more severe manifestations (Chahrour and Zoghbi, 2007). Among the atypical forms, the preserved speech variant (Zappella variant) mainly caused by MECP2 mutations, the congenital variant (Rolando variant) closely related to mutations in the FOXG1 gene, and the early seizures variant (Hanefeld variant) associated with mutations in CDKL5 gene (Operto et al., 2019; Tascini et al., 2022) are described. Males with MECP2 mutations present further diagnostic and classification challenges. Early reports had suggested that the mutation in MECP2 leads to embryonic male lethality or early postnatal death. However, subsequent studies have clarified the clinical features of affected males proving that the severity of clinical involvement in males with MECP2 mutations is remarkably broad, ranging from neurodevelopmental disorders to neonatal encephalopathy associated with early death (Neul et al., 2018; Ramirez et al., 2020). Female patients with Rett syndrome may survive into middle age and older. However, patients have a sudden and unexpected death rate of 26%, much higher than equivalent age healthy individuals, and generally due to respiratory infection, cardiac instability, respiratory failure, and sudden death in epilepsy (SUDEP) (Kyle et al., 2018; Singh et al., 2020).

## Autonomic dysfunction in Rett syndrome: Clinical manifestations and putative mechanisms

Autonomic dysregulation is widespread in patients with RTT posing a clinical challenge for treating and improving the quality of life of these patients (Singh et al., 2020). Patients exhibit considerable inter-individual differences with autonomic dysfunction that can result in various manifestations, such as breathing dysrhythmias when awake, temperature dysregulation, sweating, peripheral vascular changes, cold and blue lower extremities, enteric (gastrointestinal) changes, and cardiac abnormalities, which require a personalized therapeutic approach (Weese-Mayer et al., 2006; Halbach et al., 2012; Singh and Santosh, 2018).

The autonomic dysfunction may affect cardiorespiratory function in these patients and might in part explain the higher relative incidence of sudden death in this population. Nearly 20% of patients have prolongation of the corrected QT interval (QTc) (McCauley et al., 2011), suggesting repolarization abnormality that can predispose to unstable fatal cardiac rhythm and increase the risk of sudden cardiac death. Notably, specific MECP2 mutations (R255*, T158M) are more likely to predispose to QTc prolongation (Crosson et al., 2017; Clark et al., 2020). Furthermore, severe sinus bradycardia (Madan et al., 2004) and possible lethal ventricular tachyarrhythmia (Guideri and Acampa, 2005) have been reported. In addition, De Felice et al. (2012) highlighted a subclinical myocardial dysfunction in patients with RTT. The authors found a mild to moderate decrease in systolic and diastolic left and right ventricles longitudinal function in both typical and atypical RTT compared to age-matched controls (De Felice et al., 2012).

Regarding respiratory manifestations, more than 80% of patients exhibit breathing dysfunction during their lifespan (Ramirez et al., 2020). Many types of breathing abnormalities have been recognized and categorized into two groups: hyperventilation (shallow, fast, and/or forceful breathing) and breath-holding (apnea, Valsalva and/or apneustic breathing) (Tarquinio et al., 2018). On average, breath-holding occurs before hyperventilation and could trigger events that will determine intermittent hypoxia, leading to overactivity in chemosensitive carotid bodies, which could worsen hyperventilation episodes between breath-holds (Ramirez et al., 2020).

The mechanism of autonomic dysfunction in RTT has not been fully elucidated yet. The crucial mechanism seems to be the impaired sympathetic and parasympathetic balance (Julu and Witt Engerström, 2005; Kumar et al., 2017). The two components of the ANS are essential in regulating visceral functions to maintain the body’s homeostasis and enable it to adapt to external and internal stimuli through interconnected regulatory mechanisms operating at different central and peripheral levels (Montano et al., 2009).

**FIGURE 1:**
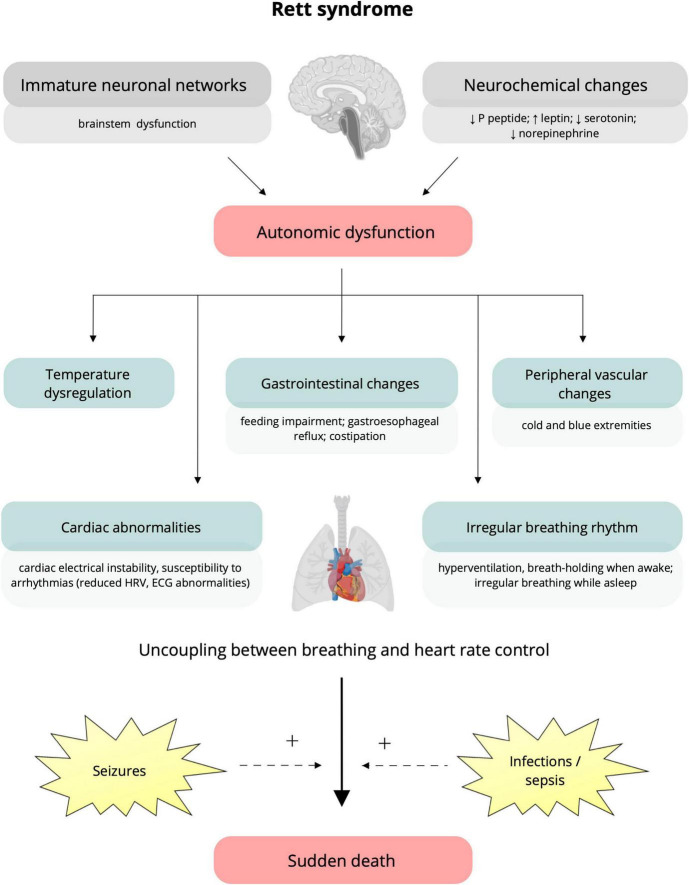
Putative underlying mechanisms and clinical manifestations of autonomic dysfunction in Rett syndrome. Immature brain networks (specifically brainstem immaturity) and neurochemical alterations (lower levels of P peptide, serotonin, norepinephrine, and increased leptin) (on the top) are assumed to be responsible for the autonomic dysfunction characteristic of Rett syndrome (Singh et al., 2020). Clinical manifestations include temperature dysregulation, alterations in the gastrointestinal system (e.g., feeding impairment, gastroesophageal reflux, and constipation), and peripheral vascular changes resulting in cold and blue extremities (Halbach et al., 2012; Singh et al., 2020). To note, autonomic dysfunction can impact cardiorespiratory function, causing irregular breathing, cardiac electrical instability, and increased susceptibility to arrhythmias, represented by reduced heart rate variability and ECG abnormalities (prolonged QTc, T-wave abnormalities) (Weese-Mayer et al., 2006; Singh and Santosh, 2018; Singh et al., 2020). The occurrence of seizures and infections can increase the underlying autonomic impairment and precipitate cardiorespiratory dysfunction, leading to sudden death (Singh et al., 2020). HRV, heart rate variability; ECG, electrocardiogram; QTc, corrected QT interval; +, potentiation.

The current literature indicates the role of immature neuronal networks and brainstem immaturity, which would induce altered cardiorespiratory autonomic control, irregular breathing, increased cardiac electrical instability and susceptibility to arrhythmias. Therefore, the intervention of events such as seizures and infections could increase this underlying autonomic impairment and precipitate cardiorespiratory dysfunction that can result in sudden death (Singh et al., 2020; Figure 1). Also, neurochemical changes are among possible mechanisms that could contribute to autonomic dysfunction in RTT. Studies on a mouse model reveal irregular breathing rhythm explained by altered medullary levels of norepinephrine and serotonin (Viemari, 2005; Weese-Mayer et al., 2006). These data from experimental models agree with serotonin transporter disorders reported in RTT patients (Weese-Mayer et al., 2006). Furthermore, elevated values of leptin, a substance playing a central role in cardiovascular systems by interaction with the sympathetic nervous system, were found in patients with RTT. Finally, substance P peptide was found to be lower in the cerebrospinal fluid of patients with RTT, and autopsy samples showed reduced substance P levels in the areas of the brainstem required to supplement cardiorespiratory responses (Singh et al., 2020).

Among putative mechanisms of autonomic dysfunction, the breathing rhythm, arterial baroreflex sensitivity, and cardiac vagal modulation maybe involved (Julu et al., 2001). Indeed, sympathetic responses during hyperventilation are poorly regulated, indicating a lack of integrative inhibition within cardiorespiratory neurons, and the oscillation in blood pressure during breath-holding indicates a lack of parasympathetic containment of the sympathetic system (Julu et al., 2001). Based on these data, subsequent the cardiorespiratory responses in both wakefulness and sleep have been investigated. Weese-Mayer et al. (2006) assessed respiratory inductance through chest/abdomen plethysmography and ECG during wakefulness, and found more irregular breathing pattern, higher breathing frequency and increased inspiratory flow than controls. Concerning cardiac regulation, the heart rate response to breath-holds was increased in patients versus controls. These results reveal a dysregulation of the heart rate response to breath-holds, and breaths preceding breath-holds. Subsequently, the authors characterized the cardiorespiratory phenotype during the night-time. Comparing patients with controls, breathing was found to be more irregular, the respiratory cycle length shorter with increasing respiratory rate, and the R-R interval briefer. These observations allowed the authors to conclude that RTT breathe more irregularly, faster, and deeper, with higher heart rates during non-breath hold breathing/apnea while asleep, indicating a cardiorespiratory autonomic dysfunction during night and day (Weese-Mayer et al., 2008). This uncoupling between breathing and heart rate control may offer insight into the mechanisms that make patients with RTT more vulnerable to sudden death (Weese-Mayer et al., 2008).

Further information can be gained by directly investigating the role of MECP2 gene mutation in cardiorespiratory dysfunction. Functionally, MECP2 is a nucleoprotein that binds to methylated DNA and is believed to play an important role in the regulation of gene transcriptions (Fan and Hutnick, 2005). Literature data suggest that MECP2 regulates the gene expression responsible for maintaining normal cardiac development and cardiomyocyte structure. Specifically, MECP2 is expressed in the developing heart, and overexpression of MECP2 in the heart causes embryonic lethality with cardiac septum hypertrophy (Alvarez-Saavedra et al., 2010). Loss of MECP2 been shown to lead to dysregulation of endogenous cardiac genes and structural alterations in the myocardium (Hara et al., 2015).

Studies in MECP2 global and targeted knockout mice revealed respiratory irregularities with frequent periods of prolonged respiratory cycles resembling breath-holds in RTT patients (Ramirez et al., 2020). Prolongation of the QTc interval has been described in a primate MECP2 mutant model of RTT (Chen et al., 2017). Moreover, spontaneous cardiac rhythm abnormalities, bradycardic events, sinus pauses, atrioventricular block, premature ventricular contractions, non-sustained ventricular arrhythmias, and increased heart rate variability have been reported in mice lacking MECP2 function (Herrera et al., 2016) in which death was associated with spontaneous cardiac arrhythmias and complete conduction block. In addition, Cheng et al. showed prolonged ventricular action potential from ventricular myocytes isolated from Mecp2*^Null/Y^* mouse hearts (Cheng et al., 2022).

To note, Bissonnette et al. (2007) characterized cardiac autonomic function in MECP2 deficient mice evaluating baseline blood pressure, pulse interval (PI) and HRV indexes (standard deviation of PI, range encompassing 90% of PIs, and standard deviation of adjacent PIs) founding similar results in MECP2^+/+^ and MECP2^+/–^ animals. Spectral analysis of mean arterial pressure and pulse interval (PI) in the frequency domain showed similar relative power. The administration of atropine or propranolol and elevation in ambient temperature resulted in changes that did not differ between the two groups (Bissonnette et al., 2007).

Moreover, Hara et al. (2015) did not demonstrate cardiac functional abnormalities or QT prolongation in MeCP2 null mice. It should be explained, in part, due to methodological discrepancies such as differences in the animal age and type of experimental model. For instance, the MeCP2 null mice should be not the best model to outline the pathophysiological mechanisms of QT prolongation because of the substantial differences in the profile of cardiac ion channels between humans and rodents (Bissonnette et al., 2007; Cheng et al., 2022). In a recent study (Cheng et al., 2022), two pairs of human induced pluripotent stem cells (iPSCs) from a female patient with RTT were used [isogenic wildtype (MeCP2wildtype) and mutant (MeCP2mutant)]. Also, they were differentiated into functional cardiomyocytes to investigate the potential mechanisms of QT prolongation and sudden death in RTT. The authors demonstrated that MeCP2 mutant induced pluripotent stem-cell-derived cardiomyocytes (iPSC-CMs) exhibited prolonged action potential and increased frequency of spontaneous early after polarization. RNA sequencing analysis revealed up-regulation of various Wnt family genes in MeCP2 mutant iPSC-CMs. These results suggest that a loss of function MeCP2 mutation can alter the electrophysiological properties of human cardiomyocytes even though there were no significant differences in the structure and morphology of CMs derived from wildtype and mutant RTT iPSCs (Cheng et al., 2022). From a clinical point of view, these intrinsic cardiac abnormalities may underlie QT prolongation and sudden death in RTT. However, there were no significant differences in the structure and morphology of CMs derived from wildtype and mutant RTT iPSCs.

Taken together, these findings suggest that the specific role of MECP2 in the autonomic dysfunction in RTT is not yet fully elucidated and may be more complex than explored so far.

Understanding the neural mechanisms of autonomic dysfunction and its correlation with sudden death is essential for patient care and experimental evidence for an association between propensity for lethal arrhythmias and signs of either increased sympathetic or reduced vagal activity has spurred efforts to develop quantitative markers of autonomic activity (Guideri et al., 2001). These putative mechanism and clinical manifestations underlying the autonomic dysfunction in RTT are summarized in Figure 1.

## Heart rate variability analysis as a method for assessing cardiac autonomic control in Rett syndrome

Cardiac autonomic regulation may be non-invasively assessed by the heart rate fluctuations. HRV analysis is widely used and is a reliable tool for cardiac autonomic assessment in physiological and pathological conditions, offering insight into the functioning of sympathetic and parasympathetic components of the nervous system (Task Force of the European Society of Cardiology and the North American Society of Pacing and Electrophysiology, 1996). HRV is closely regulated by the central autonomic network involving brainstem, prefrontal cortex, limbic system, and other higher-order brain structures. To note, a shift of the autonomic balance toward a sympathetic predominance has been associated with a higher risk of mortality (Tsuji et al., 1996; Malliani and Montano, 2002), and highlighted as a prognostic marker of adverse clinical outcome in cardiopulmonary (Tobaldini et al., 2020, 2021), neurodegenerative (Carandina et al., 2022; Lazzeri et al., 2022) and autoimmune (Rodrigues et al., 2019, 2022) diseases. In recent years, the study of HRV has found increasingly wide applications, opening up fascinating research fields in various neurological and neuropsychiatric disturbances. HRV has been employed to investigate patients with epilepsy, founding consistently lower interictal HRV, thus suggesting a shift in autonomic balance toward sympathetic dominance (Myers et al., 2018b). Of note, awake HRV was found to be lower in subjects who subsequently had SUDEP (Myers et al., 2018a). HRV reduction has also been found in children with autism spectrum disorder; interestingly, the association between more severe repetitive behaviors and HRV decrease has been documented (Patriquin et al., 2019; Thapa et al., 2021). In the context of neurodevelopmental disorders, patients with attention-deficit/hyperactivity disorder (ADHD) show reduced vagally-mediated HRV after a task demand (Robe et al., 2019). In addition, a relative cardiac autonomic imbalance (a lower parasympathetic modulation) has also been found in pediatric patients with Prader-Willi syndrome (Brito et al., 2021). Concerning psychiatric disorders, patients with schizophrenia and bipolar disorder exhibit reductions in HRV, which have been linked to disease severity (Benjamin et al., 2021).

As for the RTT, some studies assessed cardiac ANS function by HRV analysis. Most are case-control (RTT vs. healthy control subjects) studies, involving female subjects undergoing electrocardiogram (ECG) recordings during wakefulness.

Guideri et al. (1999) reported that RTT patients have a reduced HRV total power compared to an age-matched control group. Distinguishing patient groups according to the disease stage (stages II, III, and IV), the authors found that the reduction was more evident across clinical stages (Guideri et al., 1999). In a subsequent study, Guideri et al. (2001) compared females with classic RTT, AR patients with preserved language, and age-matched healthy controls, noting that patients with classic form had a significantly lower HRV total power than variant RTT patients and controls (Guideri et al., 2001). Interestingly, there were no differences between controls and AR patients. The sympatho-vagal balance was higher in females with classic RTT than females RTT with preserved speech and controls. Finally, the authors showed that the LF index of HRV was further reduced after 1 year, per the possibility that progressive decline of their autonomic disarray develops as a function of the time and clinical stage (Guideri et al., 2001).

To investigate the role of MECP2 mutation, HRV was evaluated in two siblings with intellectual disability and progressive spastic paraparesis and their mildly affected sister with A140V mutation in MECP2 gene. The analysis of spectral components of HRV showed a shift sympatho-vagal balance to a sympathetic predominance and vagal withdrawal, suggesting an autonomic dysfunction in these patients (Dotti et al., 2004). Notably, the relationships between HRV and neurochemical changes were evaluated to better understand the mechanisms underlying autonomic dysfunction. First, a correlation between augmented sympatho-vagal balance (i.e., LF/HF ratio) and low plasma serotonin levels was found, suggesting that cardiac dysautonomia in RTT could be linked to serotonergic dysfunction (Guideri et al., 2004). Furthermore, Acampa et al. (2008) found a relationship between plasma levels of leptin and LF/HF. In particular, leptin levels correlated positively with LF and negatively with HF suggesting a putative role of leptin in the genesis of cardiac dysautonomia (Acampa et al., 2008).

More recent studies confirm differences between HRV in patients with RTT and healthy controls but also provide insights for future studies by evaluating correlations with clinical severity or genotype. Kumar and colleagues reported a reduced vagal-mediated HRV and an increased sympatho-vagal balance in patients with RTT compared to controls, suggesting a cardiac autonomic dysfunction. Notably, no significant correlation between disease severity scores and HRV have been found (Kumar et al., 2017). However, studies in the current literature have been conducted almost exclusively during the daytime, except for a few recent research.

Carroll and colleagues investigated diurnal variation in autonomic regulation analyzing HRV through nighttime and daytime recordings using a wearable physiology recording system which captured ECG, RIP and SpO_2_ data (Carroll et al., 2020). First, time-domain HRV indexes (vagal modulation) was reduced in RTT compared to controls. From the 24-h analysis, HF power showed a consistent pattern of reduction in RTT, and LF/HF ratio was higher in RTT, meaning a shift toward the sympathetic modulation to the heart. Circadian analysis indicated an alteration in the acrophase and HRV modulation amplitude in the 24 h between RTT and controls. Furthermore, the authors performed an analysis by genotype, considering MECP2 variants as early truncation (ET), late truncation (LT), and missense (MS), founding more consistent differences between controls and patients with ET MECP2 mutations demonstrating that HRV indexes are also sensitive to phenotypic differences arising from different subclasses of MECP2 variants.

A recent study by Singh et al. (2022) measured HRV using non-invasive wearable sensors. First, the authors observed that heart rate decreases with age and is lower at night across all ages studied. However, they found no changes in sympathetic and parasympathetic indices with age. The indices were higher during the day when compared to the night. These findings suggest that patients with Rett are less adaptable to autonomic changes during the night (Singh et al., 2022). Table 1a summarizes the current literature of HRV assessment in RTT patients.

**TABLE 1:** Main characteristics of the studies focused on HRV analysis included in the review.

References	Country	Design	Sample description		Aim	Measurements	Results
			Sample size	Age (yrs)			
Guideri et al., 1999	IT	Case-control	54, RTT patients 28, healthy controls	10 ± 5.5 9.71 ± 4.6	To evaluate HRV as measure of sudden death risk	ECG (12-lead, 10 min) Spectral method (fast Fourier Transformation)	Lower HRV power spectrum in RTT patients
Guideri et al., 2001	IT	Case-control	74, classic RTT patients 10, RTT with preserved speech patients 40, healthy controls	10.2 ± 5.3 9.4 ± 6.8 10.2 ± 4.9	To evaluate HRV as measure of sudden death risk, comparison between classic and atypical forms	ECG (12-lead, 10 min) Spectral method (fast Fourier Transformation)	Lower HRV power spectrum in classic RTT patients than healthy controls and atypical RTT HRV further reduced after one year
Guideri et al., 2004	IT	Case-control	28, RTT patients (10 without AEDs, 18 with AEDs)	7.25 ± 3.4	To evaluate the relation between cardiac dysautonomia and plasma serotonin levels	ECG (12-lead, 10 min) Spectral method (fast Fourier Transformation)	In untreated RTT patients, plasma serotonin levels correlated with the sympathovagal balance
Dotti et al., 2004	IT	Case series	2, males, 1, female MECP2 gene mutation, no RETT	34 and 29, 44	To analyze HRV in subjects with the MECP2 gene mutation and no RTT	ECG Spectral method (fast Fourier Transformation)	HRV spectral components showed significant imbalance in sympathetic tone
Guideri et al., 2005	IT	Case-control, randomized	22, RTT patients 10 treated with ALC 12 not treated with ALC (controls)	6.3 ± 4.3 6.3 ± 4.0	To investigate the effects of long-term treatment with ALC on HRV	ECG (12-lead, 10 min) Spectral method (fast Fourier Transformation) Assessment at baseline and 6-18-month follow-up	Increase in total power, VLF, and LF spectral variables in treated patients
Acampa et al., 2008	IT	Case series	32, RTT patients	12.1 ± 6.3	To evaluate link between plasma leptin levels and sympathetic activity in RTT patients	ECG (12-lead, 10 min) Plasma leptin levels were determined by the enzyme-linked immunosorbent assay method	Correlation between plasma levels of leptin and LF/HF (positive correlation with LF and negative with HF) suggesting a role of leptin in the genesis of cardiac dysautonomia
Kumar et al., 2017	IND	Case-control	24, RTT patients 24, healthy controls	9.06 ± 3.4 9.75 ± 3.13	To evaluate autonomic functions in RTT patients, correlation of clinical severity score with HRV	ECG Spectral method (fast Fourier Transformation) Evaluation of time and frequency domain	Significantly reduced HRV in RTT patients, sympathovagal imbalance No significant correlation between severity scores and HRV
Carroll et al., 2020	US	Case-control	47, RTT patients and controls	2–7	Assessment of diurnal variation in autonomic regulation	ECG, RIP and SpO2 data using wearable physiology recording system during nighttime and daytime Analysis of HR, SDNN, RMSSD, pNN50, TriInd, SampEn Correlation with genotype	Reduced HRV in RTT, higher LF: HF ratio Analysis by genotype: patients with early truncating mutations are most different from controls
Gualniera et al., 2021	UK	Case series	10, females 4 treated with buspirone, 2 sertraline, 1 gabapentin, 3 drug free	11.87 ± 4.97	To evaluate HRV as measure of treatment response of EBAD symptoms	HRV obtained from inter beat interval (IBI) data based on raw blood volume pulse (BVP)	HRV analysis not associated with clinical outcomes
Singh et al., 2022	UK	Observational	45, RTT patients	16.46 ± 9.29	To investigate autonomic features of HRV, to determine any patterns HRV indices across the age range	Wearable sensor device; blood volume pulse (BVP) to determine HRV indices at day and night	Decrease in HR with age; HR lower at night at all ages; no change in sympathetic and parasympathetic indices with age; higher indices during the day than at night

RTT, Rett syndrome; HRV, heart rate variability; yrs, years, ECG, electrocardiogram; min, minutes; AEDs, antiepileptic drugs; ALC, acetyl-L-carnitine; EBAD, emotional, behavioural and autonomic dysregulation; RIP, respiratory inductance plethysmography; HR, heart rate; SDNN, SD of normal-to-normal intervals; RMSSD, root mean square of successive R-R interval differences; pNN50, percentage of successive interval differences greater than 50 ms; TriInd, triangle index with 8 ms histogram bins; SampEn, non-linear sample entropy measure with a two- sample match window and a noise threshold of 0.2 of the sample SD. IT, Italy; UK, United Kingdom; IND, India; US, United States.

## Heart rate variability analysis as a marker for potential therapeutic target in Rett syndrome

Cardiac autonomic control should be a potential therapeutic target in RTT, and in this scenario, HRV analysis could be employed as a marker of response after pharmacological treatment. Guideri et al. (2005) investigated the effects of long-treatment with acetyl-L-carnitine (ALC) on HRV, evaluating 22 girls with RTT at baseline and 6–18-month follow-up. Ten girls were randomized to undergo ALC treatment (50 mg/kg/day), while 12 girls formed the age-matched control group. In patients with RTT without therapy, the HRV total power was significantly reduced at 6–18-month follow-up. While in patients treated with ALC, a significant increase in HRV total power was observed. These results suggest that ALC augments the cardiac vagal modulation opposing the sympathetic overactivity in RTT, highlighting a potential therapeutical pathway to reduce the risk of cardiac arrhythmias and sudden death (Guideri et al., 2005).

Heart rate variability analysis was also used as a marker to monitor the effects of treatment with buspirone, sertraline, and gabapentin. Gualniera et al. (2021) performed a pilot study monitoring the response of Emotional, Behavioral, and Autonomic Dysregulation (EBAD) to these treatments. However, HRV metrics did not reveal significant patterns associated with clinical outcomes. As stated by the authors, this finding could be affected by the small sample size (*n* = 10) and methodological problems, given that the reliability and comparability of HRV results were affected by markedly variable signal quality (Gualniera et al., 2021).

## Conclusion and future perspectives

The data reported in this review support the hypothesis of widespread autonomic dysfunction as a pathophysiological hallmark in patients with Rett syndrome. In general, RTT patients present a shift of the sympatho-vagal balance toward a sympathetic predominance and a vagal withdrawal compared to age-matched healthy controls. However, data on correlations with different genotypes and clinical features are still scanty and invite further studies in this field.

Most studies were conducted during the day in wakefulness, with few exceptions, although the presence of autonomic dysfunction during the night has been reported as well. Considering the critical role of the ANS in the modulation of cardiac function during different sleep stages (Tobaldini et al., 2013), it would be interesting to evaluate HRV dynamics during sleep in RTT. Moreover, non-linear methods of HRV analysis could be more sensitive to detect specific physiopathological patterns in RTT, as well as occur in some disease conditions (Rodrigues et al., 2019, 2022; Tobaldini et al., 2020). Further studies might be conducted to investigate putative mechanisms underlying specific reflexes of ANS in RTT, such as baroreflex and chemoreflex.

Lastly, as to the evidence of a sympathetic predominance, cardiac autonomic control could be a target of treatments. Recent studies have explored various approaches to restore the shifted sympatho-vagal balance. In particular, the possibility of targeting the parasympathetic branch of the ANS through vagus nerve stimulation (VNS) has emerged in recent years (Carandina et al., 2021). However, the invasiveness and cost of the surgical procedure and the possible adverse events generally associated with implantation limit the clinical applicability of VNS and restrict its use for research purposes. In particular, the most feared adverse events are surgery-related, such as infections and dysrhythmias; while adverse events associated with stimulation, including cough, paresthesia, pain, and voice alteration, generally decrease in prevalence over time (Ben-Menachem et al., 2015). Interestingly, the more recent transcutaneous stimulation of the auricular branch of the vagus nerve (tVNS) is considered an effective non-invasive alternative, without the side effects of surgical procedures but with similar efficacy to the invasive device with promising applications in various conditions such as cardiovascular disorders (Guideri et al., 2004; Ben-Menachem et al., 2015; Murray et al., 2016). The cardiac and respiratory issues of patients with Rett syndrome could be thought to possibly contraindicate the employment of VNS. However, some knowledge is provided by the use of vagus nerve stimulation in the treatment of refractory epilepsy. Patients with RTT who underwent VNS displayed some side effects such as decreased appetite and coughing/choking on food, similar to patients with different diagnoses but not an exacerbation of their baseline breathing irregularities or cardiac complications (Wilfong and Schultz, 2006; Xie et al., 2022). In addition, the non-invasive tVNS, not employed in patients with this syndrome yet, could minimize typical side effects of invasive stimulation and increase tolerability. Interestingly, the prolonged QTc was a predictor of VNS responsiveness in adults with major depressive disorder (MDD). MDD patients with longer QTc at baseline showed a higher level of improvement on mood scale following 12 and 24 months of VNS therapy (Longpré-Poirier et al., 2020). It suggests that prolonged QTc at baseline may elect candidates who can most effectively respond to VNS. The application of neuromodulation approaches warrants further investigations in RTT.

## Author contributions

RC, ET, GDR, DG, MV, LC, ND, ET-D, LF, AC, GP, LN, and NM: conception of the work. RC, ET, and GDR: writing—original draft preparation. RC, ET, GDR, DG, MV, LC, ND, EDG, ET-D, LF, AC, GP, LN, and NM: writing—review and editing. LN and NM: supervision and funding acquisition. All authors read and agreed to the published version of the manuscript.
